# 3,3′-(1-Oxopropane-1,3-di­yl)bis­(1,3-thia­zolidine-2-thione) chloro­benzene hemisolvate

**DOI:** 10.1107/S1600536813003292

**Published:** 2013-02-13

**Authors:** Christine Franzel, Andrew Purdy, Ray J. Butcher

**Affiliations:** a Naval Research Laboratory, Chemistry Division, code 6100, 4555 Overlook Av, SW, Washington, DC 20375, USA; b Howard University, Chemistry Dept, Washington, DC 20059, USA

## Abstract

The title compound, C_9_H_12_N_2_OS_4_·0.5C_6_H_5_Cl, which contains two 1,3-thia­zolidine-2-thione rings, is a by-product of the synthesis of 3-acryloyl-1,3-thia­zolidine-2-thione. The dihedral angle between these rings is 79.95 (9)°, with both rings displaying a twisted conformation. The twist angle of the amide group is 5.6 (1)°. In the crystal, the molecules are linked into [001] chains by C—H⋯O interactions. The chloro­benzene solvent mol­ecule was found to show unresolvable disorder about a centre of inversion and its contribution to the scattering was removed with the SQUEEZE option in *PLATON* [Spek (2009 ▶). *Acta Cryst.* D**65**, 148–155].

## Related literature

For *N*-substituted 1,3-thia­zolidine-2-thione and for further synthetic details, see: Evans & Thomson (2005 ▶). For the defin­ition of amide twist angles, see: Yamada *et al.* (1993 ▶). For details of the use of SQUEEZE, see: van der Sluis & Spek (1990 ▶).
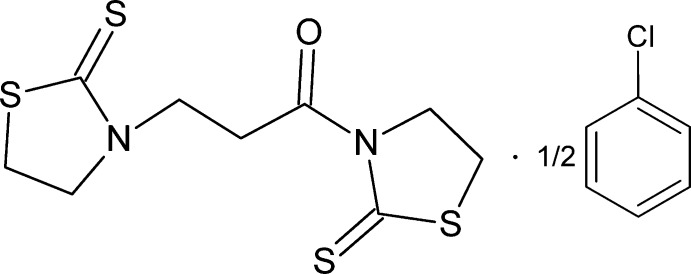


## Experimental

### 

#### Crystal data

C_9_H_12_N_2_OS_4_·0.5C_6_H_5_Cl
*M* *_r_* = 348.72
Monoclinic, 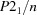

*a* = 8.59506 (18) Å
*b* = 9.4435 (2) Å
*c* = 18.2640 (4) Å
β = 92.614 (2)°
*V* = 1480.90 (6) Å^3^
*Z* = 4
Cu *K*α radiation
μ = 6.68 mm^−1^
*T* = 123 K
0.45 × 0.25 × 0.14 mm

#### Data collection

Agilent Xcalibur (Ruby, Gemini) diffractometer
Absorption correction: multi-scan [*CrysAlis PRO* (Agilent, 2012 ▶), based on expressions derived by Clark & Reid (1995 ▶)] *T*_min_ = 0.757, *T*_max_ = 1.000
5329 measured reflections
2971 independent reflections
2486 reflections with *I* > 2σ(*I*)
*R*_int_ = 0.029

#### Refinement

*R*[*F* ^2^ > 2σ(*F* ^2^)] = 0.044
*wR*(*F* ^2^) = 0.116
*S* = 1.05
2971 reflections
145 parameters
H-atom parameters constrained
Δρ_max_ = 0.44 e Å^−3^
Δρ_min_ = −0.32 e Å^−3^

### 

Data collection: *CrysAlis PRO* (Agilent, 2012 ▶); cell refinement: *CrysAlis PRO*; data reduction: *CrysAlis PRO*; program(s) used to solve structure: *SHELXS97* (Sheldrick, 2008 ▶); program(s) used to refine structure: *SHELXL97* (Sheldrick, 2008 ▶); molecular graphics: *SHELXTL* (Sheldrick, 2008 ▶); software used to prepare material for publication: *SHELXTL*.

## Supplementary Material

Crystal structure: contains datablock(s) I, global. DOI: 10.1107/S1600536813003292/hb7002sup1.cif

Structure factors: contains datablock(s) I. DOI: 10.1107/S1600536813003292/hb7002Isup2.hkl

Supplementary material file. DOI: 10.1107/S1600536813003292/hb7002Isup3.mol

Supplementary material file. DOI: 10.1107/S1600536813003292/hb7002Isup4.cml

Additional supplementary materials:  crystallographic information; 3D view; checkCIF report

## Figures and Tables

**Table 1:** Hydrogen-bond geometry (Å, °)

*D*—H⋯*A*	*D*—H	H⋯*A*	*D*⋯*A*	*D*—H⋯*A*
C9—H9*A*⋯O1^i^	0.99	2.55	3.340 (4)	137

Symmetry code: (i) 
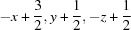
.

**Fig. 1.**
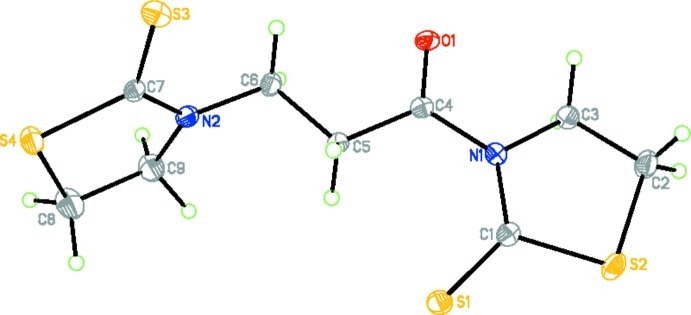
Diagram of the title compound showing 50% displacement ellipsoids.

**Fig. 2.**
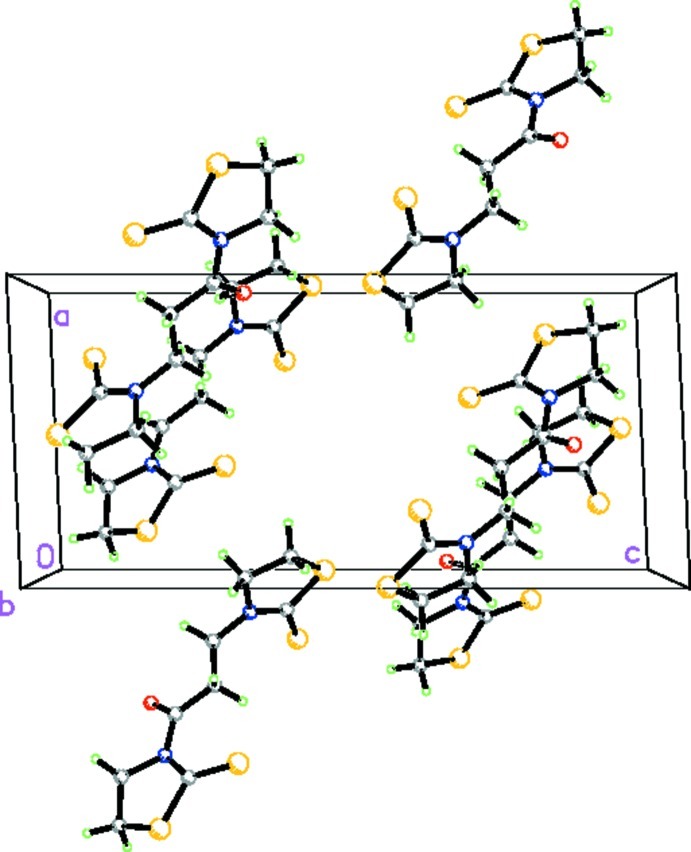
Molecular packing of C9H12N2OS4, viewed down the b axis.

## supplementary crystallographic information

### Comment

The title compound, 3,3'-(1-oxopropane-1,3-diyl)bis(1,3-thiazolidine-2-thione), is a by-product of the synthesis of 3-acryloyl-1,3-thiazolidine-2-thione. The formation of the title compound results from a nucleophilic attack of the nitrogen of excess 1,3-thiazolidine-2-thione, on the terminal alkene of 3-acryloyl-1,3-thiazolidine-2-thione.

The crystallographic data show both of the thiazolidine-2-thione rings display a twist conformation; C2 and C3 are displaced 0.269 (4) Å and -0.070 (4) Å respectively from the mean N1 C1 S1 S2 plane, while C8 and C9 are displaced 0.118 (4) Å and -0.175 (4) Å respectively from the mean N2 C7 S3 S4 plane. These two planes form a dihedral angle of 79.97 (9)°. The amide present in the title compound is nearly flat, with a twist angle about the C4–N1 bond of 5.6 (1)° (calculated according to the definition given by Yamada 1993).

Figure 2 shows the molecular packing for C_9_H_12_N_2_OS_4_, modified using the SQUEEZE function. The void in the center of the unit cell contains a disordered molecule of chlorobenzene, the recrystallization solvent used in this experiment, lying on a center of inversion.

### Experimental

The procedure for the synthesis of 3-acryloyl-1,3-thiazolidine-2-thione was modeled after that of Evans & Thomson (2005). Briefly, 1,3-thiazolidine-2-thione (2.27 g, 19.0 mmol) and Et_3_N (1.93 g, 18.9 mmol) in CH_2_Cl_2_ (50 ml) were added to a round-bottom flask with an egg-shaped stir bar and a pinch of phenothiazine inhibitor. The flask was placed in an EtOH/dry ice bath (-78°C) and kept under N_2_. Acryloyl chloride (1.71 g, 18.9 mmol) in CH_2_Cl_2_ (10 mL) was added dropwise to the flask over 5 minutes. The reaction mixture was slowly warmed to room temperature and stirred for 1 h. The yellow product mixture was then washed with H_2_O, and the aqueous layer was extracted twice with CH_2_Cl_2_. The combined organic layers were extracted twice with saturated NaHCO_3_, once more with H_2_O, dried (MgSO_4_), filtered, and concentrated. One grain of CuCl_2_ was added to the flask as polymerization inhibitor. The reaction mixture was poured into CHCl_3_, and the title compound precipitated as a light-yellow solid. The solid title compound was recrystallized from chlorobenzene solution to give almost colorless prisms.

NMR Spectrum of title compound (CH_2_Cl_2_ solution): ^1^H 4.539, 4.162, 4.044, 3.697, 3.302, 3.275 (t, 2H); ^13^C 202.26 (C=S), 196.85 (C=S), 172.06 (C=O), 57.73, 56.09, 44.90, 35.63, 28.43, 27.65 (CH_2_). IR Spectrum (film on KBr): 2988(vw), 2930(w), 2929(vw), 2890(vw), 2854(vw) (C—H), 1690 (s, C=O), 1488 (*s*), 1453 (w), 1424 (w), 1365 (*m*), 1316 (*s*), 1278 (*s*), 1224 (*s*), 1157(*s*), 1050 (*m*), 1027 (w), 995 (w), 884 (w).

### Refinement

H atoms were placed in calculated positions with C—H = 0.99 Å for the CH_2_ H atoms, and refined using riding model, with *U*_iso_(H) = 1.2Ueq(C,*N*). There was a molecule of chlorobenzene located on an inversion center but disordered over multiple conformations. After unsuccessful attempts to model this SQUEEZE was used to remove its contribution (Van der Sluis & Spek, 1990).

### Crystal data

**Table:**

C_9_H_12_N_2_OS_4_·0.5C_6_H_5_Cl	*F*(000) = 724
*M**_r_* = 348.72	*D*_x_ = 1.564 Mg m^−^^3^
Monoclinic, *P*2_1_/*n*	Cu *K*α radiation, λ = 1.54184 Å
*a* = 8.59506 (18) Å	Cell parameters from 2930 reflections
*b* = 9.4435 (2) Å	θ = 4.7–75.6°
*c* = 18.2640 (4) Å	µ = 6.68 mm^−^^1^
β = 92.614 (2)°	*T* = 123 K
*V* = 1480.90 (6) Å^3^	Prism, colorless
*Z* = 4	0.45 × 0.25 × 0.14 mm

### Data collection

**Table:**

Agilent Xcalibur (Ruby, Gemini) diffractometer	2971 independent reflections
Radiation source: Enhance (Cu) X-ray Source	2486 reflections with *I* > 2σ(*I*)
Graphite monochromator	*R*_int_ = 0.029
Detector resolution: 10.5081 pixels mm^-1^	θ_max_ = 75.8°, θ_min_ = 4.9°
ω scans	*h* = −9→10
Absorption correction: multi-scan [*CrysAlis PRO* (Agilent, 2012), based on expressions derived by Clark & Reid (1995)]	*k* = −11→10
*T*_min_ = 0.757, *T*_max_ = 1.000	*l* = −22→20
5329 measured reflections	

### Refinement

**Table:**

Refinement on *F*^2^	Primary atom site location: structure-invariant direct methods
Least-squares matrix: full	Secondary atom site location: difference Fourier map
*R*[*F*^2^ > 2σ(*F*^2^)] = 0.044	Hydrogen site location: inferred from neighbouring sites
*wR*(*F*^2^) = 0.116	H-atom parameters constrained
*S* = 1.05	*w* = 1/[σ^2^(*F*_o_^2^) + (0.0574*P*)^2^ + 1.0183*P*] where *P* = (*F*_o_^2^ + 2*F*_c_^2^)/3
2971 reflections	(Δ/σ)_max_ = 0.001
145 parameters	Δρ_max_ = 0.44 e Å^−^^3^
0 restraints	Δρ_min_ = −0.32 e Å^−^^3^

### Special details

**Table:**

Experimental. CrysAlisPro, Agilent Technologies, Version 1.171.35.21 (release 20-01-2012 CrysAlis171 .NET) (compiled Jan 23 2012,18:06:46) Analytical numeric absorption correction using a multifaceted crystal model based on expressions derived by R.C. Clark & J.S. Reid. (Clark, R. C. & Reid, J. S. (1995). Acta Cryst. A51, 887-897)
Geometry. All e.s.d.'s (except the e.s.d. in the dihedral angle between two l.s. planes) are estimated using the full covariance matrix. The cell e.s.d.'s are taken into account individually in the estimation of e.s.d.'s in distances, angles and torsion angles; correlations between e.s.d.'s in cell parameters are only used when they are defined by crystal symmetry. An approximate (isotropic) treatment of cell e.s.d.'s is used for estimating e.s.d.'s involving l.s. planes.
Refinement. Refinement of *F*^2^ against ALL reflections. The weighted *R*-factor *wR* and goodness of fit *S* are based on *F*^2^, conventional *R*-factors *R* are based on *F*, with *F* set to zero for negative *F*^2^. The threshold expression of *F*^2^ > σ(*F*^2^) is used only for calculating *R*-factors(gt) *etc*. and is not relevant to the choice of reflections for refinement. *R*-factors based on *F*^2^ are statistically about twice as large as those based on *F*, and *R*- factors based on ALL data will be even larger.

### Fractional atomic coordinates and isotropic or equivalent isotropic displacement parameters (Å^2^)

**Table:**

	*x*	*y*	*z*	*U*_iso_*/*U*_eq_	
S1	0.38391 (7)	0.58687 (8)	0.30163 (4)	0.02748 (18)	
S2	0.16697 (8)	0.60275 (8)	0.17409 (4)	0.02960 (19)	
S3	0.75412 (7)	0.06152 (7)	0.39778 (4)	0.02613 (18)	
S4	1.01178 (8)	0.25235 (8)	0.44956 (4)	0.02741 (18)	
O1	0.5444 (2)	0.2367 (2)	0.15272 (10)	0.0247 (4)	
N1	0.3858 (2)	0.4161 (2)	0.17961 (11)	0.0194 (4)	
N2	0.8680 (3)	0.2837 (2)	0.32473 (12)	0.0232 (5)	
C1	0.3274 (3)	0.5256 (3)	0.22011 (14)	0.0210 (5)	
C2	0.1553 (4)	0.4708 (4)	0.10302 (18)	0.0398 (8)	
H2A	0.0692	0.4039	0.1114	0.048*	
H2B	0.1363	0.5160	0.0545	0.048*	
C3	0.3092 (3)	0.3939 (3)	0.10594 (14)	0.0235 (5)	
H3A	0.2922	0.2915	0.0971	0.028*	
H3B	0.3762	0.4309	0.0676	0.028*	
C4	0.5170 (3)	0.3302 (3)	0.19528 (14)	0.0193 (5)	
C5	0.6201 (3)	0.3555 (3)	0.26305 (13)	0.0180 (5)	
H5A	0.5621	0.3338	0.3073	0.022*	
H5B	0.6522	0.4561	0.2653	0.022*	
C6	0.7637 (3)	0.2609 (3)	0.26076 (14)	0.0254 (5)	
H6A	0.7308	0.1604	0.2590	0.030*	
H6B	0.8196	0.2815	0.2158	0.030*	
C7	0.8692 (3)	0.2009 (3)	0.38392 (13)	0.0195 (5)	
C8	1.0683 (4)	0.4060 (4)	0.39771 (18)	0.0404 (8)	
H8A	1.0306	0.4937	0.4206	0.048*	
H8B	1.1831	0.4111	0.3959	0.048*	
C9	0.9945 (4)	0.3887 (3)	0.32101 (18)	0.0355 (7)	
H9A	0.9523	0.4804	0.3030	0.043*	
H9B	1.0732	0.3558	0.2869	0.043*	

### Atomic displacement parameters (Å^2^)

**Table:**

	*U*^11^	*U*^22^	*U*^33^	*U*^12^	*U*^13^	*U*^23^
S1	0.0230 (3)	0.0325 (4)	0.0272 (3)	0.0043 (3)	0.0034 (2)	−0.0095 (3)
S2	0.0241 (3)	0.0368 (4)	0.0281 (3)	0.0123 (3)	0.0033 (2)	−0.0017 (3)
S3	0.0224 (3)	0.0275 (3)	0.0290 (3)	−0.0045 (2)	0.0071 (2)	−0.0041 (3)
S4	0.0287 (3)	0.0288 (4)	0.0242 (3)	−0.0057 (3)	−0.0048 (2)	0.0034 (3)
O1	0.0292 (9)	0.0204 (9)	0.0244 (9)	0.0041 (8)	0.0012 (7)	−0.0059 (7)
N1	0.0190 (9)	0.0212 (10)	0.0180 (9)	−0.0019 (8)	0.0027 (7)	0.0001 (8)
N2	0.0277 (11)	0.0201 (11)	0.0216 (10)	0.0042 (9)	−0.0003 (8)	−0.0007 (8)
C1	0.0167 (11)	0.0217 (12)	0.0252 (12)	−0.0014 (9)	0.0066 (9)	0.0029 (10)
C2	0.0347 (16)	0.0485 (19)	0.0350 (16)	0.0171 (15)	−0.0120 (13)	−0.0150 (14)
C3	0.0253 (12)	0.0265 (13)	0.0189 (12)	0.0016 (11)	0.0019 (9)	−0.0003 (10)
C4	0.0189 (11)	0.0175 (11)	0.0218 (12)	−0.0024 (9)	0.0053 (9)	0.0025 (9)
C5	0.0201 (11)	0.0170 (11)	0.0170 (11)	0.0000 (9)	0.0024 (9)	0.0004 (9)
C6	0.0257 (12)	0.0271 (14)	0.0228 (12)	0.0075 (11)	−0.0038 (10)	−0.0027 (11)
C7	0.0157 (10)	0.0224 (12)	0.0205 (11)	0.0063 (9)	0.0019 (9)	−0.0045 (9)
C8	0.0521 (19)	0.0308 (16)	0.0373 (17)	−0.0188 (14)	−0.0080 (14)	0.0101 (13)
C9	0.0443 (17)	0.0287 (15)	0.0327 (15)	−0.0086 (13)	−0.0064 (13)	0.0085 (12)

### Geometric parameters (Å, º)

**Table:**

S1—C1	1.650 (3)	C2—H2B	0.9900
S2—C1	1.741 (3)	C3—H3A	0.9900
S2—C2	1.799 (3)	C3—H3B	0.9900
S3—C7	1.673 (3)	C4—C5	1.508 (3)
S4—C7	1.744 (3)	C5—C6	1.526 (3)
S4—C8	1.811 (3)	C5—H5A	0.9900
O1—C4	1.206 (3)	C5—H5B	0.9900
N1—C1	1.379 (3)	C6—H6A	0.9900
N1—C4	1.408 (3)	C6—H6B	0.9900
N1—C3	1.486 (3)	C8—C9	1.520 (4)
N2—C7	1.334 (4)	C8—H8A	0.9900
N2—C6	1.456 (3)	C8—H8B	0.9900
N2—C9	1.475 (4)	C9—H9A	0.9900
C2—C3	1.508 (4)	C9—H9B	0.9900
C2—H2A	0.9900		
			
C1—S2—C2	94.31 (13)	C4—C5—H5A	109.8
C7—S4—C8	93.47 (14)	C6—C5—H5A	109.8
C1—N1—C4	129.1 (2)	C4—C5—H5B	109.8
C1—N1—C3	115.8 (2)	C6—C5—H5B	109.8
C4—N1—C3	114.9 (2)	H5A—C5—H5B	108.3
C7—N2—C6	123.1 (2)	N2—C6—C5	111.2 (2)
C7—N2—C9	116.9 (2)	N2—C6—H6A	109.4
C6—N2—C9	119.4 (2)	C5—C6—H6A	109.4
N1—C1—S1	130.4 (2)	N2—C6—H6B	109.4
N1—C1—S2	110.76 (19)	C5—C6—H6B	109.4
S1—C1—S2	118.87 (15)	H6A—C6—H6B	108.0
C3—C2—S2	106.8 (2)	N2—C7—S3	127.0 (2)
C3—C2—H2A	110.4	N2—C7—S4	111.9 (2)
S2—C2—H2A	110.4	S3—C7—S4	121.13 (15)
C3—C2—H2B	110.4	C9—C8—S4	106.6 (2)
S2—C2—H2B	110.4	C9—C8—H8A	110.4
H2A—C2—H2B	108.6	S4—C8—H8A	110.4
N1—C3—C2	108.4 (2)	C9—C8—H8B	110.4
N1—C3—H3A	110.0	S4—C8—H8B	110.4
C2—C3—H3A	110.0	H8A—C8—H8B	108.6
N1—C3—H3B	110.0	N2—C9—C8	107.9 (2)
C2—C3—H3B	110.0	N2—C9—H9A	110.1
H3A—C3—H3B	108.4	C8—C9—H9A	110.1
O1—C4—N1	117.9 (2)	N2—C9—H9B	110.1
O1—C4—C5	121.4 (2)	C8—C9—H9B	110.1
N1—C4—C5	120.7 (2)	H9A—C9—H9B	108.4
C4—C5—C6	109.3 (2)		
			
C4—N1—C1—S1	−3.8 (4)	N1—C4—C5—C6	−172.5 (2)
C3—N1—C1—S1	−177.4 (2)	C7—N2—C6—C5	97.3 (3)
C4—N1—C1—S2	177.3 (2)	C9—N2—C6—C5	−92.1 (3)
C3—N1—C1—S2	3.7 (3)	C4—C5—C6—N2	179.0 (2)
C2—S2—C1—N1	8.0 (2)	C6—N2—C7—S3	−1.3 (4)
C2—S2—C1—S1	−170.96 (19)	C9—N2—C7—S3	−172.1 (2)
C1—S2—C2—C3	−16.8 (3)	C6—N2—C7—S4	177.92 (19)
C1—N1—C3—C2	−16.4 (3)	C9—N2—C7—S4	7.1 (3)
C4—N1—C3—C2	169.0 (2)	C8—S4—C7—N2	4.2 (2)
S2—C2—C3—N1	20.6 (3)	C8—S4—C7—S3	−176.58 (19)
C1—N1—C4—O1	177.7 (2)	C7—S4—C8—C9	−13.2 (3)
C3—N1—C4—O1	−8.6 (3)	C7—N2—C9—C8	−17.1 (4)
C1—N1—C4—C5	−2.6 (4)	C6—N2—C9—C8	171.7 (3)
C3—N1—C4—C5	171.0 (2)	S4—C8—C9—N2	18.3 (3)
O1—C4—C5—C6	7.2 (3)		

### Hydrogen-bond geometry (Å, º)

**Table:**

*D*—H···*A*	*D*—H	H···*A*	*D*···*A*	*D*—H···*A*
C9—H9*A*···O1^i^	0.99	2.55	3.340 (4)	137

Symmetry code: (i) −*x*+3/2, *y*+1/2, −*z*+1/2.
